# Shuangdan Mingmu Capsule for Diabetic Retinopathy: A Systematic Review and Meta-Analysis of Randomized Controlled Trials

**DOI:** 10.1155/2023/4655109

**Published:** 2023-01-25

**Authors:** Jiaan Du, Yingqi Mao, Youhua Xu, Kai Qu, Aiwei Han, Qibiao Wu, Lili Yu

**Affiliations:** ^1^Faculty of Chinese Medicine, Macau University of Science and Technology, Macau 999078, China; ^2^The State Key Laboratory for Quality Research in Chinese Medicines of the Macau University of Science and Technology, Macau 999078, China; ^3^Shaanxi Provincial Hospital of Chinese Medicine, Xi'an 710003, China; ^4^Guangdong-Hong Kong-Macao Joint Laboratory for Contaminants Exposure and Health, Guangdong University of Technology, Guangzhou, Guangdong, China; ^5^Zhuhai MUST Science and Technology Research Institute, Zhuhai, Guangdong, China

## Abstract

**Objective:**

To systematically evaluate the efficacy and safety of the Shuangdan Mingmu capsule in the treatment of diabetic retinopathy (DR).

**Methods:**

Common Chinese and English databases, including PubMed, Medline, Embase, VIP, Wanfang, and the Chinese National Knowledge Infrastructure (CNKI), were searched from their inception to May 31, 2022. According to the Cochrane Handbook, two reviewers independently evaluated and collected data on the included studies. Meta-analysis was performed by RevMan software 5.4.

**Results:**

Seven trials with a total of 835 patients were included. The clinical effectiveness rate was defined as the primary outcome, and the TCM symptom score, Chinese-Version Low Vision Quality of Life Questionnaire (CLVQOL) scores, macular thickness, hemorrhagic spot area, vascular endothelial growth factor levels, platelet-derived growth factor levels, and the incidence of adverse effects were the secondary outcome. The results of the meta-analysis showed that, compared with conventional medical treatment alone, the Shuangdan Mingmu capsule combined with conventional treatment could significantly improve the clinical effectiveness rate of treating DR (OR = 4.07, 95% CI (2.10, 7.89), *p* < 0.0001), and reduce the incidence of adverse reactions in DR patients (OR = 0.47, 95% CI (0.26, 0.86), *p*=0.01). In addition, other results showed that TCM symptom score(OR = −3.47, 95% CI (−3.84, −3.10), *p* < 0.00001); CLVQOL scores (OR = 23.93, 95% CI (21.37, 26.49), *p* < 0.00001); macular thickness (OR = −47.34, 95% CI (−50.67, 44.00), *p* < 0.00001); hemorrhagic spot area (OR = −0.91, 95% CI (−1.01, −0.81), *p* < 0.00001); vascular endothelial growth factor levels (OR = −45.76, 95% CI (−49.74, 41.79), *p* < 0.00001); platelet-derived growth factor levels (OR = −1.73, 95% CI (−2.15, −1.31), *p* < 0.00001).

**Conclusion:**

Compared with conventional treatment alone, the Shuangdan Mingmu capsule combined with conventional treatment is more effective and safer in the treatment of diabetic retinopathy. However, due to the limitations of the included studies, more high-quality studies are still needed to further assess the efficacy and safety of the Shuangdan Mingmu capsule in the treatment of diabetic retinopathy.

## 1. Introduction

Diabetes mellitus is a disease characterized by chronic hyperglycemia caused by metabolism disorders of sugar, fat, and protein due to insufficient insulin secretion or defective function. Diabetic retinopathy (DR) is a microangiopathy that mainly involves small blood vessels, causing microvascular occlusion, bleeding, and hemorrhage and eventually leading to retinal detachment, which seriously affects the quality of life of patients [1]. In 2019, diabetes and its complications became one of the major causes of death worldwide, and its prevalence in the world reached 10.2% [2, 3]. In 2020, the number of patients with DR in China reached 6 million, and 1.34 million were at risk of visual impairments.

At present, the conventional treatment for DR is mainly Western medicine. Calcium dobesilate, which inhibits vasoactive substances and improves microvascular circulation, is a common drug for treating DR. However, the efficacy of calcium dobesilate alone in the treatment of DR is not ideal. In China, a combination of traditional Chinese medicine (TCM) and conventional treatment has often been prescribed for DR patients with the aim of improving efficacy [4].

Shuangdan Mingmu capsule (SDMMC) is a Chinese patent medicine prepared from traditional Chinese medicines such as Nvzhenzi (*Frustus Ligustri Lucidi*) and Mohanlian (*Yerbadetajo Herb*), which have the activities of invigorating the blood and brightening the eyes, benefiting the kidney, and nourishing the liver. An increasing number of clinical trials have assessed the efficacy and safety of SDMMC combined with conventional treatment for the treatment of DR; most studies suggest that SDMMC can improve the clinical effectiveness rate (CER), TCM symptom score, and quality of life (QOL), and can also reduce adverse events [5]. However, the effects of SDMMC combined with conventional treatment for patients with DR have never been systematically evaluated.

In SDMMC, *Ligustrum lucidum* and *Moxanthus* are rich in flavonoids, which are rich in phenolic hydroxyl groups and have strong antioxidant capacity. Several studies have found that *Ligustrum lucidum* and *Moxanthus* have hepatoprotective effects on acute liver injury, and both of them can reduce the serum alanine aminotransferase (ALT) and aspartate aminotransferase (AST) levels in a dose-dependent manner in a liver injury model. It can reduce the content of malondialdehyde (MDA) in liver tissue homogenate and enhance the activity of superoxide dismutase (SOD). The combination of the two drugs can effectively improve the pathological changes in liver tissue [6].

In this study, the efficacy and safety of SDMMC combined with conventional treatment for DR patients were systematically evaluated, aiming to provide evidence for clinical practice.

## 2. Materials and Methods

### 2.1. Protocol and Registration

The present systematic review and meta-analysis of RCTs were performed following the PRISMA guidelines [7]. The protocol of this study was registered in PROSPERO with the registration number CRD42022361851 (https://crd.york.ac.uk/PROSPERO/display_record.php?RecordID=361851).

### 2.2. Criteria for Selection of Studies

#### 2.2.1. Inclusion Criteria

(1) Type of study: randomized controlled trial (RCT); (2) study population: patients with DR: refer to the clinical guidelines for DR issued by the American Academy of Ophthalmology in 2018 (Diabetic Retinopathy Preferred Practice Pattern, DR PPP); (3) experimental intervention: SDMMC combined with conventional treatment; (4) control intervention: conventional treatment. (5) outcomes: the clinical efficacy rate, TCM symptom score, CLVQOL scores, macular thickness, hemorrhagic spot area, vascular endothelial growth factor levels, platelet-derived growth factor levels, blood glucose, and adverse reactions.

#### 2.2.2. Exclusion Criteria

(1) The studies were non-RCTs; (2) patients had not been diagnosed as DR or did not have a clear diagnosis; (3) duplicate publications; (4) studies without valid outcome measures; and (5) reviews, reports of basic experiments, and reports of expert experience.

### 2.3. Search Strategy

A computerized search of English and Chinese databases, such as PubMed, Medline, Embase, VIP Database, Wanfang Database, and Chinese National Knowledge Infrastructure (CNKI), was performed. The literature search was not restricted to languages, and the search time was from the inception of these databases to May 31, 2022. Search terms included diabetic retinopathy, SDMMC, randomized controlled trial, treatment, etc. The search results were double-checked, and the references included in the literature were screened to prevent omissions.

### 2.4. Literature Screening and Data Extraction

The literature was independently screened and evaluated using Microsoft Excel by two reviewers (J.A.D and Y.Q.M). Data were extracted from the studies that met the inclusion criteria and exclusion criteria, and in case of disagreement, a third reviewer (L.L.Y, or Q.B.W) was consulted.

### 2.5. Risk of Bias Evaluation

The methodological quality of the included literature was evaluated. The risk of bias assessment tool recommended by the Cochrane Handbook was used to assess the risk of bias, which was classified into 3 levels: low risk, unclear risk, and high risk. The assessment was based on the following seven domains: random sequence generation, allocation concealment, blinding of participants and personnel, blinding of outcome assessment, incomplete outcome data, selective reporting, and other biases [8–10].

### 2.6. Statistical Analysis Methods

RevMan software 5.4 (Copenhagen: The Nordic Cochrane Centre, The Cochrane Collaboration, 2020) was applied for statistical processing. The odds ratio (OR) with 95% confidence intervals (CI) was used for count data (the clinical efficacy rate, adverse effects), and the mean difference (MD) with 95% CI was used for continuous outcomes. If *I*^2^ ≤ 50%, homogeneity was suggested, and a fixed-effects model was used; conversely, if *I*^2^ > 50%, substantial heterogeneity was suggested, and a random-effects model was used for synthetic analysis. Differences were considered statistically significant if *p* < 0.05 [11–13].

### 2.7. Publication Bias

Funnel plots were performed to examine the potential bias in the RCTs included in the meta-analysis when the number of the included RCTs was more than 10 [8, 12].

## 3. Results

### 3.1. Literature Search and Screening Results

**Figure 1:**
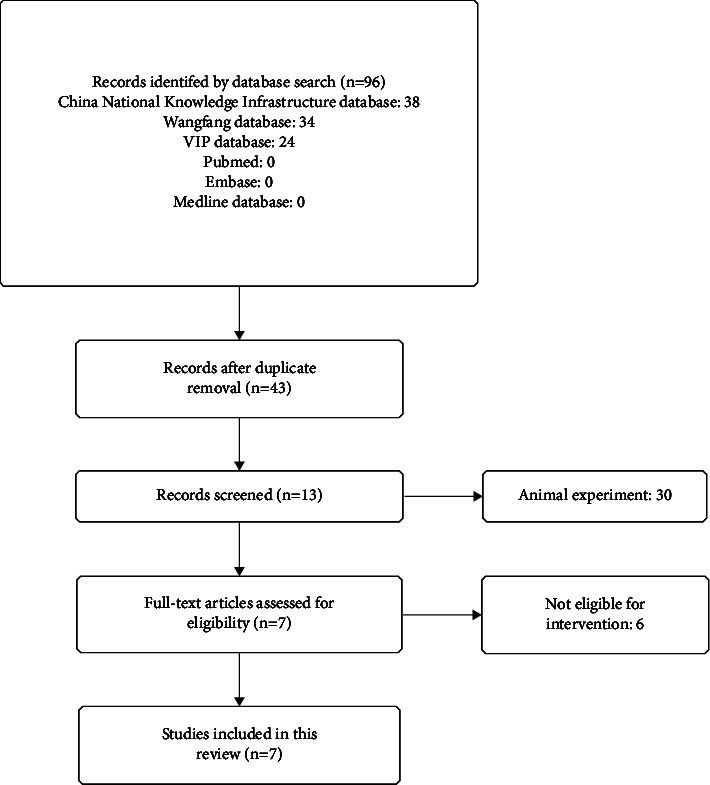
A systematic review and meta-analysis diagram of the search.

A total of 96 papers were searched; 7 studies [14–20] met the criteria and were included after eliminating duplicates and intensive reading of the abstract and full text; all the included RCTs were performed in China (Figure 1).

### 3.2. Basic Characteristics of the Included Literature

According to the inclusion and exclusion criteria, 7 RCTs with a total of 835 patients were included, including 419 cases in the test group and 416 cases in the control group [14–20]. The test group was treated with SDMMC combined with conventional treatment, and the control group was treated with conventional treatment alone (Table 1).

### 3.3. Assessment of the Quality of the Included Studies

**Figure 2:**
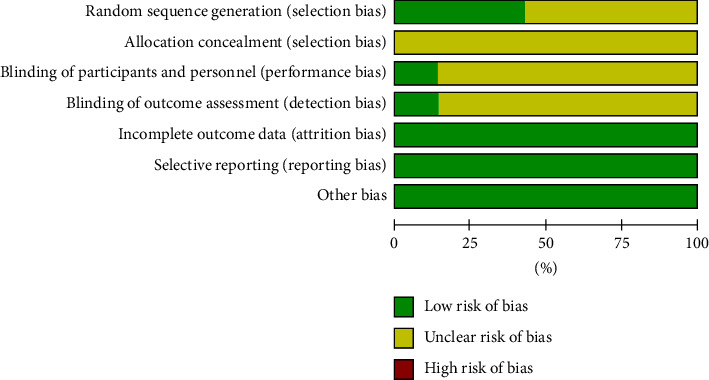
Risk of bias graph.

**Figure 3:**
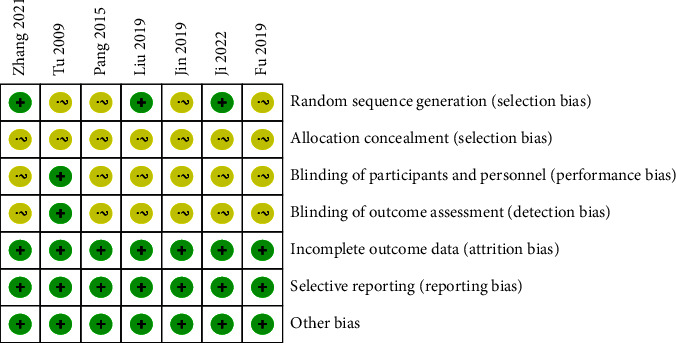
Risk of bias summary.

The baseline indicators of the seven included studies were largely consistent, but the random assignment method was unclear, and some did not mention allocation concealment and blinding. The primary outcomes of all studies were fully consistent with the expected reporting. There was no selective report bias and no other risks of bias in the included literature (Table 2, Figures 2 and 3).

### 3.4. The Clinical Effective Rate

**Figure 4:**
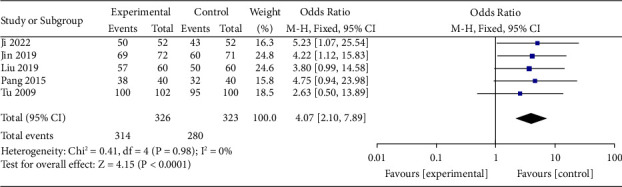
Clinical effective rate.

The clinical effectiveness rate was described in 5 studies included in this study [16–20], including 649 patients. The results of the meta-analysis showed a statistically significant difference in the clinical effectiveness rate of the test group compared with the control group (OR = 4.07, 95% CI (2.10, 7.89), *p* < 0.0001). This result indicated that SDMMC could improve the clinical effectiveness rate of treating DR (Figure 4). There was homogeneity for this outcome (*I*^*2*^ = 0%), and a fixed-effects model was used.

### 3.5. TCM Symptom Score

**Figure 5:**
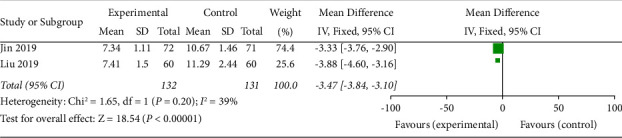
TCM symptom score.

Two studies assessed the effect of different interventions on TCM syndromes [16, 17]. Meta-analysis showed a statistically significant difference in the TCM syndrome score between the test group and the control group (OR = −3.47, 95% CI (−3.84, −3.10), *p* < 0.00001). This result indicated that the SDMMC could improve the TCM symptom score of DR patients (Figure 5). There was homogeneity for this outcome (*I*^2^ = 39%), and a fixed-effects model was used.

### 3.6. Chinese-Version Low Vision Quality of Life Questionnaire (CLVQOL) Scores

**Figure 6:**
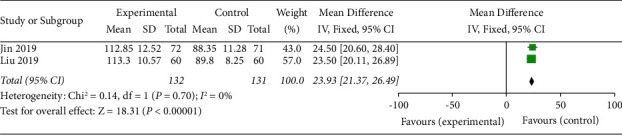
CLVQOL scores.

CLVQOL scores were described in 2 studies [16, 17]. The results of the meta-analysis showed a statistically significant difference in CLVQOL scores between the test group and the control group (OR = 23.93, 95% CI (21.37, 26.49), *p* < 0.00001). This indicated that the SDMMC improved the CLVQOL scores of DR patients (Figure 6). There was homogeneity for this outcome (*I*^2^ = 0%), and a fixed-effects model was used.

### 3.7. Macular Thickness

**Figure 7:**
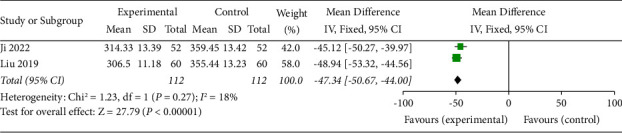
Macular thickness.

Macular thickness was described in 2 studies [16, 20]. The results of the meta-analysis showed a statistically significant difference in macular thickness between the test group and the control group (OR = −47.34, 95% CI (−50.67, 44.00), *p* < 0.00001). This result indicated that the SDMMC could reduce the macular thickness in DR patients (Figure 7). There was homogeneity for this outcome (*I*^2^ = 18%), and a fixed-effects model was used.

### 3.8. Hemorrhagic Spot Area

**Figure 8:**
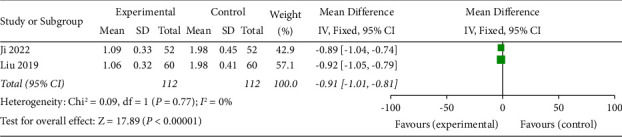
Hemorrhagic spot area.

The bleeding spot area was described in 2 included studies [16, 20]. The results of the meta-analysis showed a statistically significant difference in bleeding spot area in the test group compared with the control group (OR = −0.91, 95% CI (−1.01, −0.81), *p* < 0.00001). This result indicated that SDMMC could reduce the area of bleeding spots in DR patients (Figure 8). There was homogeneity for this outcome (*I*^2^ = 0%), and a fixed-effects model was used.

### 3.9. Vascular Endothelial Growth Factor Levels (VEGF)

**Figure 9:**
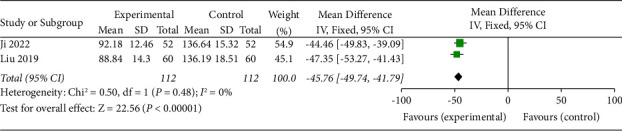
Vascular endothelial growth factor levels.

VEGF levels were described in 2 studies [16, 20]. The results of the meta-analysis showed that SDMMC could significantly downregulate VEGF levels in DR patients (OR = −45.76, 95% CI (−49.74, 41.79), *p* < 0.00001) (Figure 9). There was homogeneity for this outcome (*I*^2^ = 0%), and a fixed-effects model was used.

### 3.10. Platelet-Derived Growth Factor Levels (PDGF)

**Figure 10:**
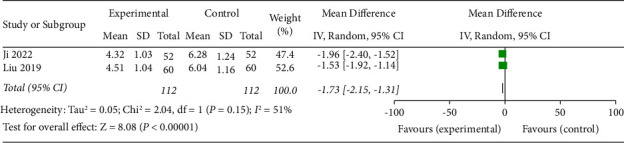
Platelet-derived growth factor levels.

PDGF levels were described in 2 studies [16, 20]. The results of the meta-analysis showed a statistically significant difference in PDGF levels in the test group compared with the control group (OR = −1.73, 95% CI (−2.15, −1.31), *p* < 0.00001). This result indicated that SDMMC significantly downregulated PDGF levels in DR patients (Figure 10). There was substantial heterogeneity for this outcome (*I*^2^ = 51%), and a random-effects model was applied.

### 3.11. Blood Glucose

Among these studies, only one study described blood glucose [15]. Comparing fasting blood glucose (FBG) and 2 h postprandial blood glucose (2 h PBG) before and after treatment between the two groups, the differences between the groups were statistically significant (*p* < 0.05). Meta-analysis was not possible for this outcome.

### 3.12. Adverse Reactions

**Figure 11:**
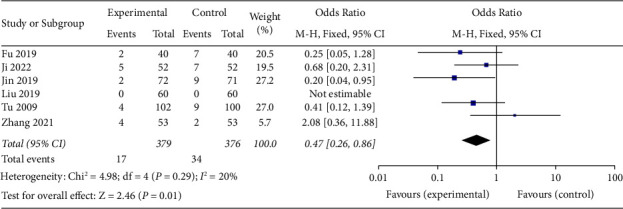
Adverse reactions.

Six studies described adverse reactions [14–17, 19, 20]. Meta-analysis using a fixed-effects model showed a statistically significant difference in adverse reactions between the groups (OR = 0.47, 95% CI (0.26, 0.86), *p*=0.01). This result indicated that SDMMC could reduce the incidence of adverse reactions in DR patients (Figure 11).

## 4. Discussion

Diabetic retinopathy is a common complication of diabetes mellitus, which mainly manifests as blurred vision, fundus exudation, and retinal edema. According to traditional Chinese medicine, DR is associated with “abrupt blindness (Bao Mang),” “dimness of vision (Shi Zhan Hun Miao),” “internal obstruction (Nei Zhang),” and “blood filling the pupil (Xue Guan Tong Shen),” etc. [21]. In 2011, the Chinese Diabetes Society summarized the etiology and pathogenesis of DR, considering yin deficiency of the liver and kidney as the underlying pathogenesis and blood stasis blocking the veins as the surface pathogenesis [22]. According to TCM theory, the functions of the SDMMC include nourishing the qi and yin of the liver and kidney, promoting blood circulation, and brightening the eyes, which work against the pathogenesis of DR.

SDMMC is derived from the combination of the Erzhi pill in the medical book “Yibian” of the Ming Dynasty and the Liuwei Dihuang pill in the “Straight Guide to Pediatric Medicine (*Xiaoer Yaozheng Zhijue*)” written by Qian Yi of the Song Dynasty. The main herbs of the formula are Nvzhenzi (*Frustus Ligustri Lucidi*) and Mohanlian (*Yerbadetajo Herb*), which can nourish the yin of the liver and kidney. In addition, Mohanlian (*Yerbadetajo Herb*) is also good at cooling the blood to stop bleeding. Shanzhuyu (*Fructus Corni*) and Shanyao (*Rhizoma Dioscoreae*) can nourish the kidney and liver and invigorate the spleen. Danshen (*Radix Salviae Miltiorrhizae*) and Sanqi (*Sanchi*) can invigorate and promote blood circulation to remove blood stasis. Mudanpi (*Cortex Moutan*), Zexie (*Rhizoma Alismatis*), and Fuling (*Poria*) have the activities of clearing the liver and draining fire, as well as drying dampness. Niuxi (*Radix Achyranthis Bidentatae*) can invigorate blood circulation, remove blood stasis, and strengthen tendons and bones; it is also good at preventing the rising of qi and blood [23, 24]. The combination of these Chinese medicinal herbs is beneficial to the kidney and liver, blood circulation, and eyesight, and is especially suitable for the treatment of diabetic retinopathy caused by yin deficiency of the liver and kidney and blood stasis [25] (Table 3).

The results of this study showed that the clinical effectiveness rate of patients in the test group was significantly higher than that of the control group; the TCM symptom score was significantly lower than that of the control group; the CLVQOL score was significantly higher than that of the control group; the macular thickness and hemorrhagic spot area were significantly smaller than those of the control group; the abovementioned differences were statistically significant (*p* < 0.05). These findings indicate that SDMMC combined with conventional treatment is effective for DR, which can improve the overall clinical efficacy rate, symptoms of patients, quality of life, and some objective signs of patients with DR.

This study also showed that the serum VEGF and PDGF levels of patients in the test group were significantly lower than those in the control group, and the differences were statistically significant (*p* < 0.05). VEGF enhances retinal capillary permeability and affects patients' visual acuity [26]. PDGF is a peptide that can be produced by a variety of cellular stimuli and can induce cell proliferation and promote extracellular matrix accumulation and monocyte-macrophage infiltration. Numerous studies have shown that PDGF is involved in the process of glucose metabolism in the diabetic state and can promote its own expression, thus forming a vicious cycle. The combination of SDMMC with conventional medicine downregulated the VEGF and PDGF levels, which might bring long-term benefits to DR patients [27, 28].

In addition, in this study, six of the seven included studies observed adverse reactions, and the meta-analysis showed that the incidence of adverse reactions in the test group was significantly lower than that in the control group, indicating that the combination of SDMMC with conventional treatment was safe and could reduce the incidence of adverse reactions in DR patients [29, 30].

There were some of the following limitations in this study: (a) the number of studies that met the inclusion criteria of this study was relatively small, all of which were written in Chinese; (b) only three of the included studies specified the randomization method, and the rest did not specify the randomization grouping method, which might have led to selective bias; (c) most of the included studies did not mention blinding, allocation concealment, follow-up, or prognosis; (d) only one study described blood glucose, meta-analysis was not possible for this outcome; (e) for all outcomes, the number of included studies was less than 10; therefore, funnel plots were not performed.

## 5. Conclusions

Compared with conventional treatment alone, the Shuangdan Mingmu capsule combined with conventional treatment is more effective and safer in the treatment of diabetic retinopathy. However, due to the limitations of the included studies, more high-quality studies are still needed to further assess the efficacy and safety of the Shuangdan Mingmu capsule in the treatment of diabetic retinopathy.

## Figures and Tables

**Table 1:** The characteristics of the included studies.

Reference	Design	Sample size (T/C)	Age (T/C)	Interventions (T/C)	Outcomes
Tu et al. 2009 [19]	RCT	102/100	1870	SDMMC + doxium/doxium	①②
Pang 2015 [18]	RCT	40/40	49.4 ± 5.7/49.6 ± 5.3	SDMMC + doxium/doxium	①
Jin and Zhang 2019 [17]	RCT	72/71	63.07 ± 8.08/62.39 ± 8.34	SDMMC + calcium dobesilate/calcium dobesilate	①②④⑤
Fu 2019 [15]	RCT	40/40	56.37 ± 11.21	SDMMC + calcium dobesilate/calcium dobesilate	③
Liu et al. 2019 [16]	RCT	60/60	57.54 ± 8.11/57.10 ± 9.26	SDMMC + calcium dobesilate/calcium dobesilate	①④⑤⑥⑦
Zhang et al. 2021 [14]	RCT	53/53	69.70 ± 2.12/69.70 ± 2.13	SDMMC + compound anisodine hydrobromide injection/compound anisodine hydrobromide injection	②
Ji and Liu 2022 [20]	RCT	52/52	56.63 ± 4.02/56.53 ± 4.09	SDMMC + calcium dobesilate/calcium dobesilate	①⑥⑦

Notes: ① clinical effective rate; ② eyesight; ③ blood glucose; ④ TCMsymptomscore; ⑤ CLVQOL scores; ⑥ macular thickness and hemorrhagic spot area; ⑦ vascular endothelial growth factor levels and platelet-derived growth factor levels RCT, randomized controlled trial; T/C, treatment group/control group.

**Table 2:** The methodologic quality of the included trials assessed using the cochrane risk of bias tool.

References	Random sequence generation	Allocation concealment	Blinding of participants and personnel	Blinding of outcome assessment	Incomplete outcome data	Selective reporting	Other bias
Tu et al. 2009 [19]	?	?	+	+	+	+	+
Pang 2015 [18]	?	?	?	?	+	+	+
Jin and Zhang 2019 [17]	?	?	?	?	+	+	+
Fu 2019 [15]	?	?	?	?	+	+	+
Liu et al. 2019 [16]	+	?	?	?	+	+	+
Zhang et al. 2021 [14]	+	?	?	?	+	+	+
Ji and Liu 2022 [20]	+	?	?	?	+	+	+

Notes: + = low risk of bias; ? = unclear risk of bias; − = high risk of bias.

**Table 3:** The Chinese medicinal herbs contained in SDMMC.

Chinese name	English name	Latin name	Family	Plant part	Processing
Nvzhenzi	*Frustus Ligustri Lucidi*	*Ligustrum lucidum* Ait.	Oleaceae	Fruit seed	Dried
Mohanlian	Yerbadetajo Herb	*Eclipta prostrate* L.	Compositae	Whole plant	Dried
Shanzhuyu	*Fructus Corni*	*Cornus officinalis* Sieb. et Zucc.	Cornaceae	Sarcocarp	Dried
Shanyao	*Rhizoma Dioscoreae*	*Dioscorea opposita* Thunb.	Dioscoreaceae	Root stock	Dried
Danshen	Radix Salviae Miltiorrhizae	*Salvia miltiorrhiza* Bunge.	Labiatae	Root stock and root	Dried
Sanqi	Sanchi	*Panax notoginseng* (Burk.) F. H. Chen	Araliaceae	Root	Dried
Mudanpi	Cortex Moutan	*Paeonia suffruticosa* Andr.	Ranunculaceae	Root bark	Dried
Zexie	Rhizoma Alismatis	*Alisma orientalis* (Sam.) Juzep	Alismataceae	Tuber	Dried
Fuling	Poria	*Poria cocos* (Schw.) Wolf	Polyporaceae	Sclerotium	Dried
Niuxi	*Radix Achyranthis Bidentatae*	*Achyranthes bidentata* Blume.	Amaranthaceae	Root	Dried

## Data Availability

The raw data utilized in this study are available publicly from VIP Database (https://qikan.cqvip.com/) , Wanfang Database (https://www.wanfangdata.com.cn/), Chinese National Knowledge Infrastructure (CNKI, https://www.cnki.net/), PubMed (https://pubmed.ncbi.nlm.nih.gov/), Medline (https://www.nlm.nih.gov/medline/index. html) and Embase (https://www.embase.com/landing?status=grey).
